# Bioactive Wollastonite-Diopside Foams from Preceramic Polymers and Reactive Oxide Fillers 

**DOI:** 10.3390/ma8052480

**Published:** 2015-05-08

**Authors:** Laura Fiocco, Hamada Elsayed, Letizia Ferroni, Chiara Gardin, Barbara Zavan, Enrico Bernardo

**Affiliations:** 1 Department of Industrial Engineering, University of Padova, Via Marzolo 9, Padova 35131, Italy; E-Mails: laurafiocco@hotmail.com (L.F.); elsisy_chem@yahoo.com (H.E.); 2 Department of Biomedical Sciences, University of Padova, Via Ugo Bassi 58/B, Padova 35131, Italy; E-Mails: letizia.ferroni@unipd.it (L.F.); chiara.gardin@unipd.it (C.G.); barbara.zavan@unipd.it (B.Z.)

**Keywords:** polymer-derived ceramics, bioactivity, wollastonite, diopside, glass-ceramic

## Abstract

Wollastonite (CaSiO_3_) and diopside (CaMgSi_2_O_6_) silicate ceramics have been widely investigated as highly bioactive materials, suitable for bone tissue engineering applications. In the present paper, highly porous glass-ceramic foams, with both wollastonite and diopside as crystal phases, were developed from the thermal treatment of silicone polymers filled with CaO and MgO precursors, in the form of micro-sized particles. The foaming was due to water release, at low temperature, in the polymeric matrix before ceramic conversion, mainly operated by hydrated sodium phosphate, used as a secondary filler. This additive proved to be “multifunctional”, since it additionally favored the phase development, by the formation of a liquid phase upon firing, in turn promoting the ionic interdiffusion. The liquid phase was promoted also by the incorporation of powders of a glass crystallizing itself in wollastonite and diopside, with significant improvements in both structural integrity and crushing strength. The biological characterization of polymer-derived wollastonite-diopside foams, to assess the bioactivity of the samples, was performed by means of a cell culture test. The MTT assay and LDH activity tests gave positive results in terms of cell viability.

## 1. Introduction

The technology of polymer-derived ceramics (PDCs) is among the most novel approaches for the synthesis and shaping of advanced ceramics. In the vast range of polymeric precursors, silicone resins are undoubtedly widely explored and exploited thanks to their low cost, large availability and easy handling [1]. The synthesis of many types of silicate ceramics can be easily achieved by the addition of metal oxide precursors, in the form of micro- or nano-sized particles. Highly phase pure ceramics can be obtained at relatively low temperatures, due to the high reactivity of the metal oxide precursors with the particularly defective network of the amorphous silica, left as a ceramic residue of oxidative decomposition of silicones [2,3].

In the field of bioceramics, Ca-silicates and Ca-Mg silicates have recently received a growing interest for their bioactivity properties, according to their ability to stimulate body tissues to repair themselves, in particular for bone ingrowth [4,5,6,7,8,9]. Silicone/fillers mixtures do not only allow one to get these peculiar bioactive formulations, but also facilitate the shaping of the ceramic components in the form of highly porous bodies, which are extremely useful, especially in the field of scaffolds for bone regeneration [10,11]. As an example, porous akermanite (Ca_2_MgSi_2_O_7_) was successfully fabricated from preceramic polymers [12], as well as porous wollastonite (CaSiO_3_) [13,14] and foamed wollastonite-diopside glass ceramic (CaSiO_3_-CaMgSi_2_O_6_) [15]. Concerning the shaping techniques, different methods can be applied, such as warm-pressing of composite powders mixed with sacrificial PMMA microbeads, evolution of CO_2_ previously entrapped in the polymer matrix by supercritical CO_2_-assisted extrusion, 3D printing of porous scaffolds from direct extrusion of preceramic pastes and foaming by water release from specific hydrated fillers [12,13,14,15].

While a high phase purity is usually achievable in binary systems derived from preceramic polymers, such as Ca-silicates, ternary systems generally imply some difficulties, due to the potential formation of undesired binary compounds instead of the expected ternary compounds. As described in a couple of previous papers, the problem may be solved by providing a liquid phase upon firing, which could promote the ionic interdiffusion, operating with specific fillers [16]. A fundamental example is that of hydrated sodium borate, also known as borax (Na_2_B_4_O_7_·10H_2_O) included in the formulations for akermanite (Ca_2_MgSi_2_O_7_) [12] and wollastonite-diopside ceramics [15]. The additive formed a borate liquid phase upon firing and helped the crystallization of the desired phases. The borate liquid phase, after cooling at room temperature, remained as a glass phase, so that the resulting product could be seen as a sort of “polymer-derived glass-ceramic”. Borax could be seen actually as a multifunctional filler, since its use in a liquid silicone could be exploited also for an abundant and uniform foaming, due to the water release associated with the dehydration reaction, occurring at only 350 °C. The cross-linking of the polymer stabilized the porosity, maintained also after the conversion of the polymer into amorphous silica and the formation of silicates [12,13,14,15]. It must be noted that Mg(OH)_2_, used as the MgO precursor for Ca-Mg silicates, may contribute to the foaming, but its impact is much lower than that of borax [12].

Although the addition of borax is undeniably significant for the obtainment of glass-ceramic samples with a specific phase assemblage and with a homogeneous cellular structure, the effect on the biocompatibility of the same samples is still controversial. Several studies highlighted a concern associated with borate bioactive glasses, due to the potential toxicity of boron released in the solution as borate ions (BO_3_)^3−^ [17,18]. As an example, the well-known borate bioglass 13-93B3 was found to be toxic to murine MLO-A5 osteogenic cells *in vitro*, above a boron threshold concentration of 0.65 mmol in the cell culture medium, while it supported the proliferation and growth of the cells below that concentration [19]. However, the same scaffolds did not show toxicity to cells *in vivo* and supported new tissue infiltration when implanted in rats [20,21,22,23]. Other boron-containing glasses are reported to be biocompatible and bioactive [24,25].

The materials described in previous papers [12,15] have a low amount of boron, but it should be remarked that boron was reasonably concentrated in the glass phase between silicate crystals. At present, the biological characterization of wollastonite-diopside porous glass-ceramics, obtained by borax addition in silicone-based mixtures, is still in progress, but it confirms the controversial impact of the specific element. In fact, dissolution studies in simulated body fluid (SBF) proved the positive behavior of the material in terms of bioactivity and ion release, while a 24 h *in vitro* cell culture test showed that the material was not suitable for cell living and proliferation.

**Figure 1:**
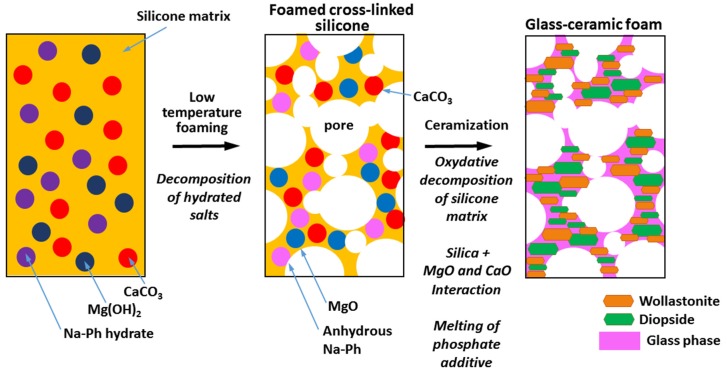
Scheme for the obtainment of wollastonite-diopside “polymer-derived glass-ceramic” foams, according to the dual role of hydrated sodium phosphate filler (Na-Ph hydrate).

In the present paper, we discuss a further development concerning highly porous wollastonite-diopside “polymer-derived glass-ceramics”, based on the replacement of borax with sodium phosphate dibasic heptahydrate (Na_2_HPO_4_∙7H_2_O), aimed at overcoming the above-described difficulties arising from the presence of boron. The selected filler, like borax, is multifunctional, *i.e.*, it contributes to both foaming and forming a liquid phase upon firing, as illustrated by Figure 1.

Like in the previously developed wollastonite-diopside ceramics [15], the addition of a further filler, in the form of powders of a glass crystallizing into wollastonite and diopside, will be discussed in order to optimize the integrity of samples. In fact, the ceramization step does not modify the macro-porosity formed in the low-temperature foaming step, but it implies the formation of micro-cracks, caused by internal stresses. The glass addition is essentially conceived to reduce the cracks, enhancing the stress relaxation operated by the liquid phase, upon firing, with no impact on foaming and phase development. 

Although preliminary, the results of a five-day cell culture test, on phosphate-modified wollastonite-diopside ceramics, indicate a good biocompatibility, independent of the glass addition.

## 2. Experimental Procedure

### 2.1. Starting Materials

Two commercially available silicones, H62C and MK (Wacker-Chemie GmbH, Munich, Germany), were considered as silica precursors, with a yield of 58 wt% and 84 wt%, respectively [2]. CaO and MgO precursors consisted of CaCO_3_ (Sigma Aldrich, Gillingham, UK) and Mg(OH)_2_ (Industrie Bitossi, Vinci, Italy), respectively, in the form of powders with a diameter below 10 µm. The amounts of silicones and precursors for CaO and MgO were calibrated in order to match the CaO-MgO-SiO_2_ molar proportion of 2-1-3, corresponding to an equimolar mixture of wollastonite (CaSiO_3_ or CaO·SiO_2_, CaO-MgO-SiO_2_ molar proportion of 1-0-1) and diopside (CaMgSi_2_O_6_ or CaO·MgO·2SiO_2_, CaO-MgO-SiO_2_ molar proportion of 1-1-2).

Sodium phosphate dibasic heptahydrate (Na_2_HPO_4_∙7H_2_O, Sigma Aldrich, Gillingham, UK) was used as additional filler. Finally, a powdered Ca/Mg-rich silicate glass with a particle size <60 µm (mean diameter ~5 μm—known as G20CaII glass [15]), was added. The chemical composition of the glass additive is reported in Table 1. The molar proportions between CaO, MgO and SiO_2_ roughly correspond to those of the desired mixture of wollastonite and diopside, with alkali oxides used as fluxes. The use of Li_2_O, in addition to Na_2_O, is in agreement with recent findings concerning the positive effect of this oxide added in formulations of bioglasses, previously involving only sodium oxide [26,27].

**Table 1:** Chemical composition of the glass additive used in silicone-based mixtures.

Composition (% mol)				
SiO_2_	CaO	MgO	Na_2_O	Li_2_O
55.3	22.0	12.0	9.0	1.7

### 2.2. Preparation of Foams

H62C was first dissolved in isopropanol (10 mL for 10 g of final ceramic) and then mixed with micro-sized fillers, including sodium phosphate, in the as-received, hydrated form (the quantity of salt was 10 wt% of the theoretical ceramic yield of the other components, corresponding to 5 wt% of anhydrous salt)*.* Selected samples included also glass powders (10 wt% of the theoretical ceramic yield of the other components). The mixing was performed under magnetic stirring, followed by sonication for 10 min, which allowed obtaining stable and homogeneous dispersions. The mixtures were poured into large glass containers and dried at 60 °C overnight.

After first drying, the mixtures were in the form of thick pastes, later manually transferred into cylindrical Al molds and then subjected to a foaming treatment at 350 °C in air for 30 min. Cylindrical samples, 10 mm in diameter and 7–8 mm in height, were obtained from the foams. The top surfaces were polished with abrasive paper. The samples (after removal from Al molds) were fired at 1100 °C for 1 h, using a heating rate of 2 °C/min.

### 2.3. Preparation of Pellets

Monolithic pellets were prepared using the MK mixed with Mg(OH)_2_ and CaCO_3_ micro-particles, anhydrous sodium phosphate (the same salt cited above, after preliminary dehydration at 450 °C, with a heating rate of 5 °C/min, for 1 h) and glass additive. MK was dissolved in isopropanol (10 mL for 10 g of final ceramic) and then mixed with the fillers. Stable and homogeneous dispersions in isopropanol were obtained using the same conditions applied for the H62C-based mixtures and left to dry overnight at 60 °C.

After drying, the silicone-based mixtures were in the form of solid fragments, later converted into fine powders by ball milling at 350 rpm for 30 min. The powders were cold-pressed in a cylindrical steel die applying a pressure of 20 MPa for 1 min, without using any additive. Specimens of 0.5 g, 16.6 mm in diameter and approximately 1.7 mm in thickness, were obtained. For comparison purposes, pellets of glass-free formulation were also prepared. The cold-pressed samples were fired at 1100 °C for 1 h, using a heating rate of 2 °C/min.

### 2.4. Cell Culture and Seeding

For cell culture studies, samples were cut to 10 mm × 10 mm × 5 mm and fixed to 48-well plates. The entire well plates where then sterilized. Human fibroblasts were seeded at a density of 4 × 10^5^ cells/piece in cDMEM, which consisted of Dulbecco’s Modified Eagle Medium (DMEM) (Lonza S.r.l., Milano, Italy), supplemented with 10 vol% fetal bovine serum (FBS) (Bidachem S.p.A., Milano, Italy) and 1 vol% penicillin/streptomycin (P/S) (EuroClone, Milano, Italy). The 3D cultures were incubated at 37 °C and 5% CO_2_ for 7 days, with media changes every 2 days. 

### 2.5. Analysis of Cell Viability 

The cell proliferation rate was evaluated after 3 and 7 days from seeding with the MTT (methylthiazolyl-tetrazolium)-based proliferation assay, performed according to the method of Denizot and Lang with minor modifications [28]. Briefly, samples were incubated for 3 h at 37 °C in 1 mL of 0.5 mg/mL MTT solution prepared in phosphate buffered saline (PBS) (Euroclone). After removal of the MTT solution by pipette, 0.5 mL of 10% DMSO in isopropanol was added to extract the formazan in the samples for 30 min at 37 °C. For each sample, absorbance values at 570 nm were recorded in duplicate on 200 μL aliquots deposited in microwell plates using a multi-label plate reader (Victor 3, Perkin Elmer, Milano, Italy).

Lactate Dehydrogenase Activity (LDH activity) was measured using a specific LDH Assay Kit (SigmaAldrich, St. Louis, MO, USA) according to the manufacturer’s instructions. All conditions were tested in duplicate. The culture medium was reserved to determine extracellular LDH. The intracellular LDH was estimated after cells lysis with the assay buffer contained in the kit. All samples were incubated with a supplied reaction mixture, resulting in a product whose absorbance was measured at 450 nm using a Victor 3 multi-label plate reader.

For SEM imaging, fibroblasts grown on samples for 3 and 7 days were fixed in 2.5% glutaraldehyde in 0.1 M cacodylate buffer for 1 h, then progressively dehydrated in ethanol. Control and treated Ti discs without cells were also examined.

### 2.6. Statistical Analysis

*t*-tests were used to determine significant differences (*p* < 0.05). Repeatability was calculated as the standard deviation of the difference between measurements. All testing was performed in SPSS 16.0 software (SPSS Inc., Chicago, IL, USA) (license of the University of Padua, Padua, Italy).

### 2.7. Characterization 

Microstructural characterizations were performed by optical stereomicroscopy (AxioCam ERc 5 s Microscope Camera, Carl Zeiss Microscopy, Thornwood, NY, USA) and scanning electron microscopy (FEI Quanta 200 ESEM, Eindhoven, The Netherlands) equipped with energy dispersive spectroscopy (EDS).

The crystalline phases were identified by means of X-ray diffraction on powdered samples (XRD; Bruker AXS D8 Advance, Bruker, Germany—CuKα radiation, 0.15418 nm, 40 kV–40 mA, 2θ = 15°–70°, step size = 0.05°, 2 s counting time), supported by data from the PDF-2 database (Powder Diffraction File, ICDD-International Center for Diffraction Data, Newtown Square, PA, USA) and the Match! program package (Crystal Impact GbR, Bonn, Germany).

The bulk density of the foams was determined from the weight-to-volume ratio, using a caliper and a digital balance. The true density of the samples was measured by means of a gas pycnometer (Micromeritics AccuPyc 1330, Norcross, GA, USA), operating with He gas on finely-milled samples. 

The crushing strength of foams was measured at room temperature, by means of an Instron 1121 UTM (Instron Danvers, MA, USA) operating with a cross-head speed of 1 mm/min. Each data point represents the average value of 5–10 individual tests.

## 3. Results and Discussion 

### 3.1. Foaming and Phase Development 

**Figure 2:**
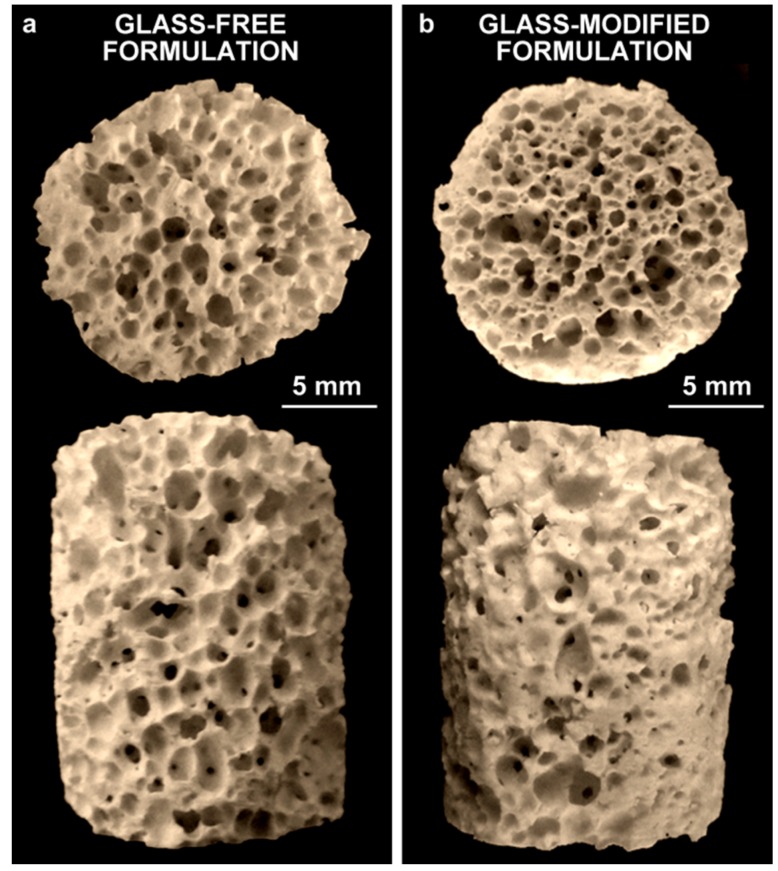
Morphology of the foams (top and side views): (**a**) glass-free formulation; (**b**) glass-modified formulation (10 wt% glass).

Figure 2a testifies to the very homogeneous foaming achieved according to the approach described in Figure 1. Many interconnections between adjacent pores were visible from both top and side views, as proof of the open porosity. The morphology of the newly obtained foams is comparable to that of previous wollastonite-diopside polymer-derived ceramics foamed by decomposition of borax, although the amount of foaming additive had to be drastically revised. The effect of 10 wt% hydrated Na-phosphate, in other words, roughly corresponded to that 3 wt% borax (samples with a lower content of phosphate salt, exhibiting a much less abundant and uniform foaming, are not discussed here for the sake of brevity) in previous experiments [15].

**Figure 3:**
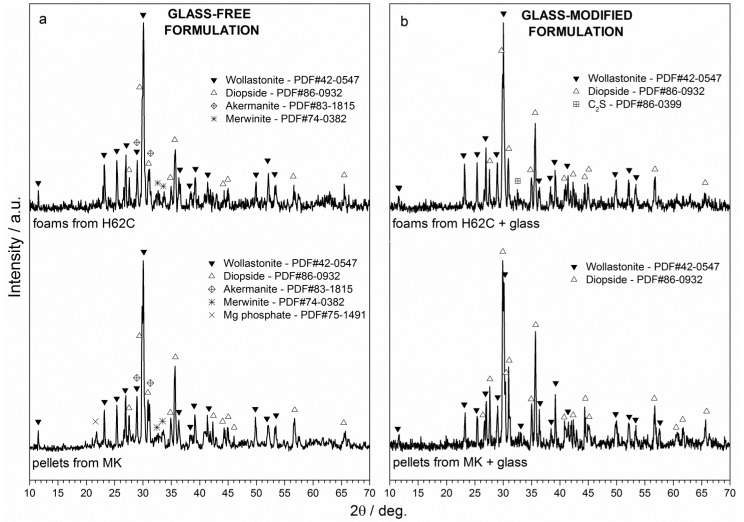
X-ray diffraction pattern of polymer-derived glass-ceramic samples (foams from H62C, pellets from MK): (**a**) glass-free formulations; (**b**) glass-modified formulations.

Like borax, the phosphate salt did not contribute to the formation of any crystal phase. In particular, Figure 3a (upper pattern) shows that the expected silicate phases, *i.e.*, wollastonite (PDF#42-0547) and diopside (PDF#86-0932), effectively formed at 1100 °C from H62C silicone and oxide precursors, with only minor traces of akermanite (PDF#83-1815) and merwinite (Ca_3_MgSi_2_O_8_; PDF#74-0382).

The similarity with the previous wollastonite-diopside foams, developed with borax, was further confirmed by the physical and mechanical data reported in Table 2. Bulk density, the amount of open porosity and crushing strength were practically identical. The crushing strength (approximately 1.5 MPa), in particular, was quite low, considering the high crystallinity inferable from the diffraction pattern (the absence of an “amorphous halo” suggested a limited amount of glass phase, mostly attributable to sodium phosphate).

**Table 2:** Physical and mechanical properties of polymer-derived wollastonite-diopside foams.

Foam Formulation	Bulk Density (g/cm^3^)	Open Porosity (%)	Crushing Strength (MPa)
H62C + fillers (borax) *	0.73 ± 0.02	77.0	1.8 ± 0.3
H62C + fillers (Na-phosphate)	0.70 ± 0.02	76.5	1.4 ± 0.1
H62C + fillers + 10 wt% glass (Na-phosphate)	0.63 ± 0.10	79.4	3.1 ± 0.7

* Data from Fiocco *et al.* [15].

**Figure 4:**
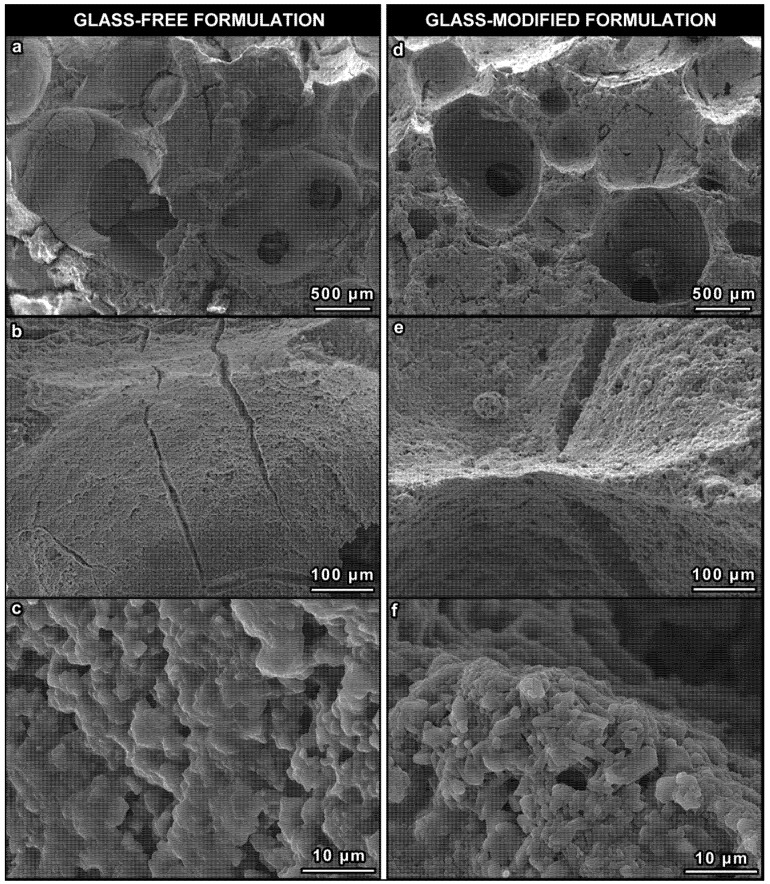
Higher magnification details of the foams: (**a**–**c**) glass-free formulation; (**d**–**f**) glass-modified formulation (10 wt% glass).

As illustrated by Figure 4a–c, the foamed samples from glass-free formulation exhibited a large number of microcracks, which could be due to the development of internal stresses upon ceramization. These stresses could be attributed to multiple factors, such as gas release from the polymer-to-ceramic conversion of silicones, decomposition of calcium carbonate (used as CaO precursor) and volume changes associated with the crystallization of silicates, visible as small granules in Figure 4c.

Despite a slightly less homogeneously distributed macro-porosity and mean diameter (Figure 2b), with respect to the samples from the glass-free formulation (Figure 2a), foams developed with glass powders as additional fillers exhibited an improvement in the structural integrity (Figure 4d–f). The viscous flow, due to the softening of glass particles, likely overlapped with that of the liquid phase offered by sodium phosphate and caused some stress relaxation. The formation of elongated crystals, shown in Figure 4f, could be seen as proof of enhanced flow. The crystals can be practically attributed only to wollastonite and diopside, considering the upper pattern of Figure 1b, showing only very small traces of dicalcium silicate (C_2_S, Ca_2_SiO_4_ or 2CaO·SiO_2_; PDF#86-0399) in addition to the well-defined peaks of the desired phases.

As reported in Table 2, both bulk density and the amount of open porosity were not affected by the glass addition. However, the glass addition was more effective, owing to the reduction of cracks, in the improvement of the mechanical strength, which increased from 1.4 ± 0.1 (for foams without glass) up to 3.1 ± 0.7 (for foams added with the 10 wt% of glass).

### 3.2. Impacts of Preceramic Polymer and Glass on Phase Development

Cell culture tests are generally easier to perform with flat samples, instead of foamed samples. For the specific purpose of preparing disc samples, H62C was replaced by MK. The solid silicone allowed an easy shaping of pellets by cold pressing of powdered silicone-fillers mixtures. The amount of MK was obviously calibrated, keeping the reference CaO-MgO-SiO_2_ molar proportion, considering the different yield of silica, compared to H62C; since no foaming was expected, sodium phosphate was used in anhydrous form.

The lower pattern of Figure 3a clearly shows that the change in the preceramic polymer had no practical impact on the phase development, except for the formation of traces of magnesium phosphate (Mg_3_P_2_O_8_; PDF#75-1491). This phosphate phase, together with akermanite and merwinite, completely disappeared in an MK-based formulation comprising glass particles, as shown in the lower pattern of Figure 3b. The “purifying” effect of the glass additive (an enhanced content of liquid phase promotes the interdiffusion), found for H62C, was confirmed in the system based on MK.

An additional discussion, concerning the phase development, can be done on the basis of semi-quantitative analysis provided by the Match! (Crystal Impact GbR, Bonn, Germany) program package, already employed for phase identification. Considering wollastonite and diopside, as a first approximation, as the only crystal phases, the program package could predict several weight ratios, reported in Table 3, corresponding to the best matching between experimental and theoretical diffraction patterns, depending on the formulation. In an ideal ceramic with wollastonite and diopside in equivalent molar amounts (molar ratio equal to one), the theoretical wollastonite/diopside weight balance would be equal to 35/65; from Table 3, we can easily note that the best agreement with the theoretical weight balance was provided by glass-modified formulation, based on both H62C and MK polymers.

As previously mentioned, the glass additive was proven to crystallize, alone, in wollastonite and diopside [15]. Considering the chemical composition (Table 1), we estimated a certain weight balance between the crystalline and amorphous phase, in the hypothesis of CaO included only in wollastonite and diopside, in equivalent molar content, as reported in Table 4. Repeating the same calculation, on the basis of the weight balances reported in Table 3, for polymer-based mixtures (Table 4, again) we can note that: (i) the amount of glass phase, in the glass-free formulation, is only slightly above that expected from the sodium phosphate additive (5 wt%); and (ii) the addition of glass did not “dilute” the crystallization, wollastonite and diopside being formed not only by polymer-filler reactions, but also by glass devitrification.

**Table 3:** Wollastonite-diopside weight balances according to the semi-quantitative X-ray diffraction analysis provided by the Match! program package.

	Formulations	Wollastonite (wt%)	Diopside (wt%)
*Theoretical*	CaO·SiO_2_ + CaO·MgO·2SiO_2_	35	65
1	H62C + fillers	56	44
2	H62C + fillers + 10 wt% glass	40	60
3	MK + fillers	49	51
4	MK + fillers + 10 wt% glass	42	58

**Table 4:** Semi-quantitative analysis of the weight balance between crystalline and amorphous phases.

	Formulations	Crystalline Phase (wt%)	Amorphous Phase (wt%)
	Pure Ca/Mg-rich glass	66	34
1	H62C + fillers	88	12
2	H62C + fillers + 10 wt% glass	98	2
3	MK + fillers	92	8
4	MK + fillers + 10 wt% glass	96	4

The calculations in Table 4 are only indicative (a more precise phase quantification, based on specific software packages, is in progress), but we can certainly say that silicone/fillers mixtures and the adopted Ca/Mg-rich glass have an intrinsic, very significant “compatibility”; one system had a great potential in supporting the other. Going back to foams from H62C, the increase of the liquid phase formed upon firing could be achieved by a simple increase of the amount of sodium phosphate additive, but with the risks of coarsening and/or viscous collapse of the cellular structure, upon firing, due to the dilution of the fraction leading to wollastonite and diopside. The glass additive represented a valid alternative, offering a “transient liquid phase”, mostly transformed in the desired crystal phases. The tests with MK, despite providing pellets for cell tests, are promising for the application of shaping techniques based on this specific polymer (foaming by release of CO_2_, embedded upon supercritical CO_2_-assisted extrusion [14]) or on MK/H62C mixtures (scaffolds from fused deposition of silicone-based pastes [13]).

### 3.3. In Vitro Biological Characterization 

**Figure 5:**
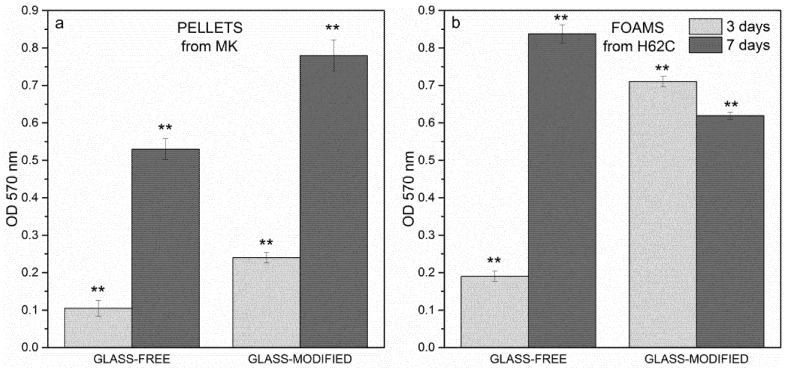
MTT assay: (**a**) pellets, 3–7 days; (**b**) foams, 3–7 days. Significant difference * (*p* < 0.05); ** (*p* < 0.01); *** (*p* < 0.001).

As previously stated, a preliminary biological study, *i.e.*, the MTT assay, was performed on MK-derived pellets. The graph in Figure 5a shows that an increase in cell viability was observed passing from Day 3–7 for both the formulations (*i.e.*, glass-free and glass-modified), implying that the fibroblast surviving at Day 3 might have duplicated and proliferated up to Day 7. Interestingly, the incorporation of glass seemed to make the pellets generally even more biocompatible. 

The successful tests on pellets stimulated the application of the MTT assay on H62C-derived foams, having a morphological organization closer to that of natural bones. As summarized in Figure 5b, at Day 3, cell viability looked higher in the glass-modified foams, as already seen in Figure 5a, while at Day 7, cells on the glass-free foams were more proliferated. From this observation, the addition of glass in the formulation of the foams did not lead to a clear improvement in cell viability at Day 7, but only contributed to increasing the biocompatibility at Day 3.

Comparing the behavior of cells seeded on pellets and on foams, with regards to glass-free formulation, the foams allowed a more extensive cell viability; concerning the glass-modified formulation, the foams showed an improvement in viability only at Day 3.

**Figure 6:**
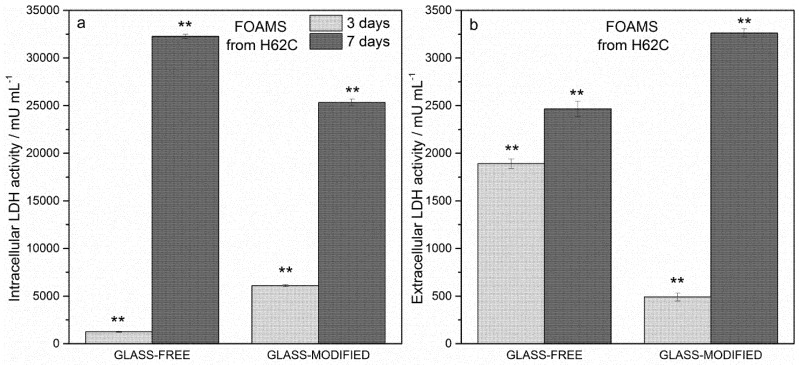
LDH activity assay. (**a**) Intracellular LDH activity, foams, 3–7 days; (**b**) extracellular LDH activity, foams, 3–7 days. Significant difference * (*p* < 0.05); ** (*p* < 0.01); *** (*p* < 0.001).

In order to overcome the controversial results of the MTT assay obtained for pellets and foams, the LDH activity assay was also performed on the cells. Figure 6a shows the intracellular LDH activity of the cells seeded on pellets: the graph proves that cells were able to produce metabolites, with improved results after seven days from seeding. As reported in Figure 6b, extracellular LDH activity was also measured on the culture medium: the graph confirms that metabolites were secreted by the same cells.

Even if the results of intracellular and extracellular LDH activity assays were not perfectly in agreement with each other, it can be observed that the incorporation of glass, which was effective in improving the mechanical behavior of the foams and the phase assemblage, was not detrimental to cell survival and proliferation.

**Figure 7:**
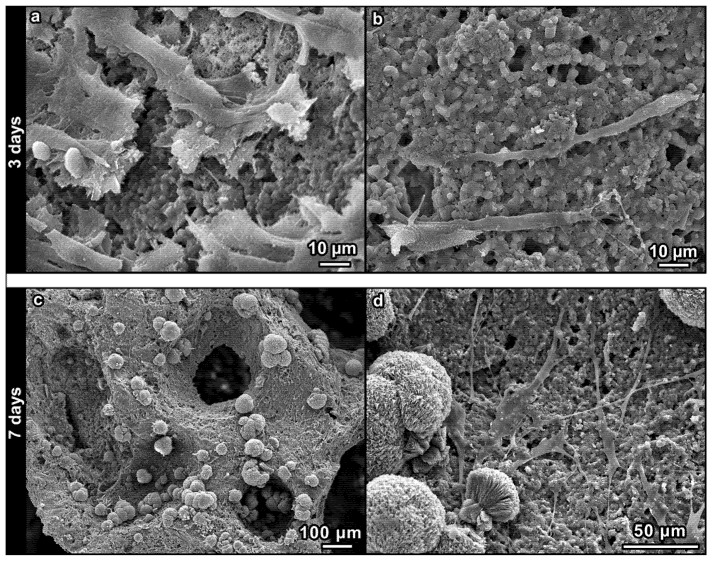
SEM images after cell culture on foams: (**a**) glass-free formulation, three days; (**b**) glass-modified formulation, three days; (**c**,**d**) glass-modified formulation, seven days.

SEM images of the foams, shown in Figure 7, were taken after three and seven days from fibroblast seeding. After three days (Figure 7a,b), fibroblasts were found to be alive and spread on the surface of the samples, of both glass-free and glass-modified formulations; in particular, they had a more elongated profile when seeding on glass-modified foams (Figure 7b). After seven days, cells had colonized the surface of the foams, still demonstrating elongated profiles, as shown in Figure 7c,d for glass-modified samples. Moreover, the formation of hydroxyapatite precipitates (nodules in Figure 7c,d) was observed, giving further evidence of the biocompatibility of the material.

## 4. Conclusions 

We may conclude that:

- Wollastonite-diopside ceramics can be fabricated by firing mixtures based on preceramic polymers, in the form of silicone resins (acting as silica sources), mixed with powdered metal oxide precursors;
- The choice of silicone polymers with different natures and chemistry (liquid H62C, solid MK) does not affect the ceramic product in terms of main phase assemblage;
- A liquid silicone can be easily foamed by water release, in turn due to the decomposition of hydrated sodium phosphate; the ceramic conversion implies the transformation of the silicone foam into a glass-ceramic foam, incorporating silicate crystals embedded in the glass phase provided by the same phosphate additive;
- The liquid phase developed upon firing can be increased by the introduction of a glass filler; the positive impact on the structural integrity of samples is not accompanied by any change in the phase assemblage, operating with a glass crystallizing itself in wollastonite and diopside;
- Both dense and foamed wollastonite-diopside ceramic samples showed positive results in terms of cell viability, according to the MTT assay and LDH activity tests; the incorporation of glass in the formulations proved not to be detrimental to cell survival and proliferation;
- While the incorporation of glass in the formulation was not crucial for viability at Day 7, it was definitively effective at improving the biocompatibility of the samples throughout the cell culture period up to Day 3.
